# Development of standard clinical endpoints for use in dengue interventional trials

**DOI:** 10.1371/journal.pntd.0006497

**Published:** 2018-10-04

**Authors:** Kay M. Tomashek, Bridget Wills, Lucy Chai See Lum, Laurent Thomas, Anna Durbin, Yee-Sin Leo, Norma de Bosch, Elsa Rojas, Kim Hendrickx, Martin Erpicum, Liane Agulto, Thomas Jaenisch, Hasitha Tissera, Piyarat Suntarattiwong, Beth Ann Collers, Derek Wallace, Alexander C. Schmidt, Alexander Precioso, Federico Narvaez, Stephen J. Thomas, Robert Edelman, João Bosco Siqueira, M. Cristina Cassetti, Walla Dempsey, Duane J. Gubler

**Affiliations:** 1 Division of Microbiology and Infectious Diseases, National Institute of Allergy and Infectious Diseases, National Institutes of Health, Rockville, Maryland, United States of America (USA); 2 Oxford University Clinical Research Unit, Hospital for Tropical Diseases, Ho Chi Minh City, Viet Nam; 3 Department of Paediatrics, University of Malaya, Kuala Lumpur, Malaysia; 4 Emergency Department, University Hospital of Martinique, Fort-de-France, Martinique; 5 Department of International Health, Johns Hopkins Bloomberg School of Public Health, Baltimore, Maryland, United States of America; 6 Institute of Infectious Diseases and Epidemiology, Tan Tock Seng Hospital, Singapore; 7 Universidad Central de Venezuela, Caracas, Venezuela; 8 Escuela de Medicina, Universidad Industrial de Santander, Bucaramanga, Colombia; 9 FWO Postdoctoral Fellow of the Life Sciences & Society Lab, Center for Sociological Research, KU Leuven and Research Associate of SPIRAL Research Center, University of Liège, Belgium; 10 SPIRAL Research Center, University of Liège, Belgium; 11 Department of Infectious Diseases, Heidelberg University Hospital, Heidelberg, Germany; 12 National Dengue Control Unit, Ministry of Health, Elvitigala Mawatha, Colombo, Sri Lanka; 13 Department of Pediatrics, Queen Sirikit National Institute of Child Health, Bangkok, Thailand; 14 Merck & Co. Inc., Kenilworth, New Jersey, United States of America; 15 Takeda Pharmaceutical Co. Ltd., Zurich, Switzerland; 16 GlaxoSmithKline plc (GSK) Vaccines, Rockville, Maryland, United States of America; 17 Division of Clinical Trials and Pharmacovigilance, Butantan Institute, São Paulo, Brazil; 18 Pediatrics Department of the Medical School of University of São Paulo, São Paulo, Brazil; 19 Infectious Diseases Unit, National Pediatric Reference Hospital, Hospital Infantil Manuel de Jesús Rivera, Managua, Nicaragua; 20 Division of Infectious Diseases, State University of New York, Upstate Medical University, Syracuse, New York, United States of America; 21 Center for Vaccine Development, University of Maryland School of Medicine, Baltimore, Maryland, United States of America; 22 Federal University of Goias, Brasilia, Brazil; 23 Emerging Infectious Diseases Programme, Duke-NUS Medical School, Singapore; University of California San Francisco, UNITED STATES

## Abstract

Dengue is a major public health problem worldwide. Although several drug candidates have been evaluated in randomized controlled trials, none has been effective and at present, early recognition of severe dengue and timely supportive care are used to reduce mortality. While the first dengue vaccine was recently licensed, and several other candidates are in late stage clinical trials, future decisions regarding widespread deployment of vaccines and/or therapeutics will require evidence of product safety, efficacy and effectiveness. Standard, quantifiable clinical endpoints are needed to ensure reproducibility and comparability of research findings. To address this need, we established a working group of dengue researchers and public health specialists to develop standardized endpoints and work towards consensus opinion on those endpoints. After discussion at two working group meetings and presentations at international conferences, a Delphi methodology-based query was used to finalize and operationalize the clinical endpoints. Participants were asked to select the best endpoints from proposed definitions or offer revised/new definitions, and to indicate whether contributing items should be designated as optional or required. After the third round of inquiry, 70% or greater agreement was reached on moderate and severe plasma leakage, moderate and severe bleeding, acute hepatitis and acute liver failure, and moderate and severe neurologic disease. There was less agreement regarding moderate and severe thrombocytopenia and moderate and severe myocarditis. Notably, 68% of participants agreed that a 50,000 to 20,000 mm^3^ platelet range be used to define moderate thrombocytopenia; however, they remained divided on whether a rapid decreasing trend or one platelet count should be case defining. While at least 70% agreement was reached on most endpoints, the process identified areas for further evaluation and standardization within the context of ongoing clinical studies. These endpoints can be used to harmonize data collection and improve comparability between dengue clinical trials.

## Author summary

Dengue is a major public health problem worldwide. Although several drug candidates have been evaluated in randomized controlled trials, none has been effective, and early recognition of severe dengue and timely supportive care remain the only means to reduce mortality. While the first dengue vaccine was recently licensed, and several other candidates are in late stage clinical trials, future decisions regarding deployment of such vaccines or therapeutics will require evidence of product safety, efficacy and effectiveness. Standard, quantifiable clinical endpoints are needed to ensure reproducibility and comparability of research findings. To address this need, we established a working group of dengue researchers, vaccine developers, and public health specialists to develop endpoints. After two working group meetings and discussions at international meetings, the Delphi methodology was used to clarify and further develop endpoints such that 70% or greater agreement was reached on most endpoint definitions including moderate and severe plasma leakage, moderate and severe bleeding, acute hepatitis and acute liver failure, and moderate and severe neurologic disease. The process identified areas for further evaluation and standardization within the context of ongoing clinical studies. The endpoints can be used to harmonize data collection and improve comparability between dengue clinical trials.

## Introduction

Dengue is a major public health problem worldwide, with an estimated 3.9 billion people at risk globally [1]. While most dengue virus (DENV) infections are asymptomatic or result in a self-limited acute febrile illness (AFI), some are life-threatening due to severe plasma leakage, severe bleeding, or, less frequently, severe organ impairment [2, 3]. Although several antiviral and immunomodulatory drug candidates have been evaluated in randomized controlled trials, none have been shown to be effective in treating dengue or preventing its severe manifestations [4, 5]. At present, early recognition of severe dengue and timely supportive care are used to reduce mortality [6–8]. However, the first dengue vaccine was recently licensed, and there are several other dengue vaccine candidates in late-stage clinical trials [9].

Future decisions regarding the use of candidate dengue vaccines and therapeutics will require evidence of product safety and efficacy, as well as demonstration of vaccine effectiveness in reducing disease burden. The current guidelines for evaluation of dengue vaccines in endemic areas recommend that the primary efficacy endpoint be prevention of virologically-confirmed dengue of any severity [10]. However, these guidelines also recommend that secondary endpoints be developed to measure outcomes such as the vaccine’s effect on severity of disease, clinical presentation, and atypical cases. In recent years, there has been increasing recognition among the dengue scientific community that establishing standard, quantifiable clinical endpoints that can be applied across a range of research activities are an important step towards ensuring that study methodology and implementation are reproducible [11–13]. Adoption of standard endpoints should greatly facilitate comparison between interventional trials conducted in diverse clinical settings, may help prevent biased reporting of selective outcomes or post hoc analyses, and could also prove useful for studies focused on understanding disease pathogenesis.

In January 2015, the National Institute of Allergy and Infectious Diseases (NIAID), part of the National Institutes of Health, and the Partnership for Dengue Control (PDC) convened an expert working group to develop standard clinical endpoints to measure moderate and severe manifestations of dengue in clinical research studies. The primary aim was to improve comparability of severity of dengue disease assessments among clinical trials. Importantly, the clinical endpoints developed as part of this endeavor are intended only for use as research tools and are not meant to replace current World Health Organization (WHO) case classification for dengue [3]. The WHO 2009 classification is intended to be broadly applicable in clinical settings and for disease surveillance, and differentiates between dengue, dengue with warning signs, and severe dengue, based on readily accessible clinical information. However, it is of limited use in clinical research because criteria for severe disease are not well-defined [14]. This could affect reproducibility between research sites, even within the same country. Further refinement and granularity is needed to develop international standards for the detailed discrimination of clinical phenotypes for use in interventional trials and pathogenesis studies [12]. In addition, severe dengue is thought to be an infrequent event, and several less severe manifestations that require medical intervention contribute disproportionately to disease burden [15]; therefore, inclusion of an intermediate endpoint, “moderately severe dengue”, was felt to be important. A group of 27 dengue researchers from academia, government, industry and public health specialists from 14 countries were invited to participate in at least one group by task: 1.) clinical endpoint development, 2.) endpoint validation, and 3.) development of a tool to characterize febrile illness experience. In this paper, we summarize the progress to date of the clinical endpoint development group.

## Methods

Twenty-two of the 27 participants volunteered to be part of the clinical endpoint development group which was divided into three work groups tasked with reviewing the literature to identify potential research endpoints from publications describing clinical dengue studies, and those developed by international medical organizations for more general use. The three groups were asked to develop endpoints for moderate and severe plasma leakage, bleeding, thrombocytopenia, liver disease, neurologic disease, and myocarditis, that were measurable, reproducible, and implementable in diverse settings. Work groups met by phone to review results from their literature review for each endpoint and discuss potential endpoint definitions. Work groups then presented and led a discussion on their proposed endpoints during a 2-day workshop with 56 participants from 16 countries, at two large international meetings, and at a final 1-day workshop with participants from the first workshop. Following these discussions, the Spiral Research Center at the University of Liège in Belgium queried participants using Delphi methodology to refine and operationalize the clinical endpoints.

The Delphi methodology is a structured tool that creates conditions that are favorable for a convergence of opinions while allowing moderators to discern points of dissent [16]. The process consisted of three rounds of inquiries, which were administered electronically using Mesydel software [17]. In the first round, participants were asked to select the best endpoint among a list of proposed endpoints to characterize disease outcomes among clinical trial participants with laboratory-confirmed dengue (Table 1). They were given the opportunity to propose another endpoint or offer ideas on how to improve their preferred endpoint in an open-ended question. Questions about endpoints were paired with questions about how to operationalize the endpoint in terms of timing and type of data to be collected including clinical laboratory tests, physical exams, and medical procedures.

**Table 1:** Proposed clinical endpoint definitions.

Proposed Clinical Endpoint Definitions Options
***Moderate plasma leakage***. **Definition A**–Participant meets either criteria: 1.) > 15% change in hematocrit, or 2.) evidence of fluid on U/S or x-ray, without hemodynamic compromise or respiratory compromise. **Definition B**–Participant must meet both criteria: 1.) > 20% change in hematocrit with or without evidence of fluid on U/S or x-ray, and 2.) no hemodynamic compromise or respiratory compromise.
***Severe plasma leakage*.** **Definition A**–Participant meets two criteria: 1.) presence of shock or respiratory compromise, and 2.) evidence of plasma leakage. **Definition B**–Participant meets all three criteria: 1.) presence of shock or respiratory compromise, and 2.) evidence of plasma leakage, AND 3.) >20% change in hematocrit (peak compared to baseline or post-recovery value).
***Respiratory compromise***. Participant has all three of following criteria: 1.) increased respiratory rate for age; 2.) sign of increased work of breathing (e.g., retractions, nasal flaring, accessory muscle use); and 3.) need for additional support to include oxygen supplementation or intubation.
***Hemodynamic instability***. Participant has either a narrow pulse pressure less than or equal to 20 mmHg OR low systolic blood pressure (SBP) for age OR drop in SBP >40 during course of the illness (e.g., in adult with chronic hypertension); AND participant has two or more signs of shock including: 1.) elevated heart rate for age, 2.) pale, cool skin, 3.) thready, weak pulse, or 4.) capillary refill delayed >2 seconds.
***Hemoconcentration*.** **Definition A**–any hematocrit reading >50%. **Definition B**–maximum hematocrit to baseline hematocrit change of >15% or >15% above normal for age and sex. **Definition C**–maximum recorded hematocrit above stabilized hematocrit at discharge >20%, or >20% above normal hematocrit for age & sex.
***Baseline hematocrit***. **Definition A**–the lowest value recorded during the acute illness, with no specification on the number or timing of the values obtained. **Definition B**–the lowest value from a minimum of 3 values obtained during the acute illness either 1.) before plasma leakage becomes apparent (day 1–3), OR 2.) after plasma leakage stabilizes (day 8 or more, a minimum of 24 hours after stopping IV fluid), OR 3.) a convalescent value obtained at follow-up within 28 days of illness onset. **Definition C**–Maximum recorded hematocrit above stabilized hematocrit at discharge >20%, or above normal hematocrit for age and sex. **Definition D**–the lowest value from a minimum of 3 values obtained during the acute illness either 1.) before plasma leakage becomes apparent (day 1–3), OR 2.) after plasma leakage stabilizes (day 8 or more, a minimum of 24 hours after stopping IV fluid), OR 3.) a convalescent value obtained at follow-up within 28 days of illness onset. If not suitable value is available, use the population mean for age and sex.
***Maximum hematocrit*.** **Definition A**–the peak value recorded during the acute illness, with no specification on the number or timing of the values obtained. **Definition B**–the peak value obtained within the time-period consistent with expected plasma leakage (day 4–8 from fever onset) with a minimum of three values obtained during the acute illness. **Definition C**–maximum recorded hematocrit above stabilized hematocrit at discharge >20%, or above normal hematocrit for age and sex. **Definition D**–the peak value obtained at any time, from a minimum of 3 values obtained during the acute illness. **Modified Definition**–highest hematocrit value recorded during the acute illness taken during the time-period consistent with expected plasma leakage (day 4–8 after fever onset) or within 48 hours of defervescence.
***Moderate bleeding***. **Definition A**–No evidence of shock or hemodynamic instability BUT intervention needed including: a) type and crossmatch even if blood is not given, or b) local intervention needed. **Definition B**–Large skin/injection site bleeding needing pressure compress. **Definition C**–Nose/gum bleeding needing local intervention (e.g., nasal or gum packing, use of adrenaline). **Definition D**–Gastrointestinal bleeding without shock or hemodynamic instability or need for blood product BUT the bleed warrants crossmatch, closer monitoring. **Definition E**–Macroscopic hematuria. **Definition F**–Vaginal bleeding with need for hormonal therapy or type and crossmatch. **Definition G**–Eye bleeding (not including conjunctival hemorrhage or petechiae) that does not affect vision.
***Severe bleeding***. **Definition A**–Any bleeding into a critical organ (e.g., CNS bleed). **Definition B**–Any bleeding leading to hemodynamic instability. **Definition C**–Any bleeding resulting in death or permanent disability (e.g., CNS bleed; intraocular bleed). **Definition D**–Any bleeding that results in need for blood transfusion AND bleed requires more intensive monitoring (ICU or HDU). [Note: prophylactic platelet transfusions or FFP given for lab values (i.e., no clinical indication) do not count and are not case defining]. **Definition E**–Any bleeding that persists after measures are taken to stop bleeding (e.g., application of pressure) AND bleed requires more intensive monitoring in a ICU or HDU.
***Moderate thrombocytopenia*.** **Definition A**–More intensive observation on a regular ward, or transfer to HDU or ICU depending on facilities available WITH decreasing trend in platelets from 100,000 to 50,000 per cubic millimeter (mm^3^) within 24 hours. **Definition B**–More intensive observation on a regular ward, or transfer to HDU or ICU depending on facilities available WITH decreasing trend in platelets from 50,000 to 20,000 mm^3^ within 24 hours. **Definition C**–More intensive observation on a regular ward, or transfer to HDU or ICU depending on facilities available WITH one platelet level between 50,000–20,000 mm^3^.
***Severe thrombocytopenia***. **Definition A**–More intensive observation on a regular ward, or transfer to HDU or ICU depending on facilities available WITH decreasing trend in platelets from 50,000 to 20,000 mm^3^ within 24 hours. **Definition B**–More intensive observation on a regular ward, or transfer to HDU or ICU depending on facilities available WITH decreasing trend in platelets from 20,000 mm^3^ downward within 24 hours. **Definition C**–More intensive observation on a regular ward, or transfer to HDU or ICU depending on facilities available WITH one platelet level less than 20,000 mm^3^. **Definition D**–More intensive observation on a regular ward, or transfer to HDU or ICU depending on facilities available WITH one platelet level less than or equal to 5,000 mm^3^.
***Moderate liver disease (acute hepatitis)*.** **Definition A**–Participant meets all 3 criteria: 1.) has an acute illness with discrete onset of signs and symptoms consistent with acute viral hepatitis (e.g., fatigue, abdominal pain, loss of appetite, intermittent nausea, vomiting, dark urine, clay-colored or light stools); 2.) alanine aminotransferase (ALT) is greater than 10 times the upper limit of normal consistent with grade 4 toxicity in the FDA 2007 toxicity tables or > = 400 U/L; and 3.) they do not meet criteria for acute liver failure (ALF) (i.e., no mental status changes and international normalized ratio (INR) <1.5). **Definition B**–Participant meets all 3 criteria: 1.) has an acute illness with discrete onset of signs and symptoms consistent with acute viral hepatitis (as defined in Definition A), 2.) ALT is greater or equal to 1000 U/L, and 3.) they do not meet criteria for ALF.
***Severe liver disease (acute liver failure)*.** **Definition A**–Participant meets all 3 criteria: 1.) has an acute clinical syndrome consistent with acute hepatitis; AND 2.) new onset change in mental status or any grade of hepatic encephalopathy that occurs after onset of hepatitis; AND 3.) new onset coagulopathy as demonstrated by an international normalized ratio (INR) > = 1.5. **Definition B**–Participant meets all 3 criteria: 1.) has an acute clinical syndrome consistent with acute hepatitis with new onset jaundice; AND 2.) new onset change in mental status or any grade of hepatic encephalopathy that occurs after onset of jaundice; AND 3.) new onset coagulopathy as demonstrated by an INR > = 1.5.
***Moderate neurologic disease***. **Definition A**–Participant meets two criteria: 1.) they have an abnormal neurologic examination; AND 2.) they DID NOT receive or were not thought to require intubation, shunting or intensive care. **Definition B**–Participant meets two criteria: 1.) they have an abnormal neurologic examination; AND 2.) neurologic involvement DOES NOT result in death or ongoing sequelae that impairs daily function. **Definition C**–Participant meets all three criteria: 1.) they have an abnormal neurologic examination; 2.) they DID NOT receive or were not thought to require intubation, shunting or intensive care; and 3.) neurologic involvement DOES NOT result in death or ongoing sequelae that impairs daily function. [NOTE: This definition will include transient mild encephalopathy defined by Glasgow Coma Score ≥12 but less than 15 for <2 days duration or other neurologic disease that cannot be defined as severe (e.g., aseptic meningitis)].
***Severe neurologic disease***. **Definition A**–Participant has an abnormal neurologic examination AND they received or were thought to require intubation, shunting or intensive care. **Definition B**–Participant has an abnormal neurologic examination AND neurologic involvement results in death or ongoing sequelae that impairs daily function. **Definition C**–Participant has an abnormal neurologic examination and: 1.) neurologic involvement that results in death or ongoing sequelae that impairs daily function; and /or 2.) they received or were thought to require intubation, shunting or intensive care. **Modified Definition C**–Participant has an abnormal neurologic examination with a Glasgow Coma Score <11 in adults or a Pediatric Glasgow Coma Scale <11 or a Blantyre coma score <3 in children; AND subject has: 1.) neurologic involvement resulting in death or ongoing sequelae that impairs daily function, and /or 2.) they received or were thought to require intubation, shunting, or intensive care or HDU level of care if an ICU is not available.
***Proposed items to be collected for dengue laboratory-confirmed cases with moderate or severe neurologic involvement*** 1.) History of clinical course including signs and symptoms and level of intervention needed; 2.) Findings from physical and neurologic examination including Glasgow Coma Scale for adults and BCS for children; 3.) Findings from CSF examination (cells, protein, glucose, etc.); 4.) Results of PCR of CSF for DENV; 5.) Findings from CSF and serum assays done to rule out co-infection with other infectious agent (i.e., offer check list to done based on circulating viruses and endemic pathogens: e.g., Zika virus, West Nile virus, Japanese encephalitis virus, YFV, malaria, etc.); 6.) Findings from brain imaging: CT scan or MRI of brain; 7.) Findings from EMG if case presents with Guillain-Barré Syndrome or other paralytic condition; and 8.) Findings from brain tissue histopathology if trial participant dies.
***Moderate myocarditis*.** **Definition A**–Participant meets either criteria: 1.) has an acute illness with discrete onset of signs and symptoms consistent with acute viral myocarditis (e.g., elevated troponin, CPK-MB, or ST2 above the laboratory ULN); or 2.) has evidence of cardiac arrhythmia and/or ST elevation > 1mm, QRS complex changes (Q waves > 0.04 sec and >0.25 of the amplitude of R wave or symmetric negative T waves. **Definition B**–Participant meets both criteria: 1.) has an acute illness with discrete onset of signs and symptoms consistent with acute viral myocarditis (e.g., elevated troponin, CPK-MB, or ST2 above the laboratory ULN); and 2.) has evidence of cardiac arrhythmia and/or ST elevation > 1mm, QRS complex changes (Q waves > 0.04 sec and >0.25 of the amplitude of R wave or symmetric negative T waves.
***Severe myocarditis***. **Definition A**–Participant meets either criteria 1 or 2, plus criteria 3: 1.) has an acute illness with discrete onset of signs and symptoms consistent with acute viral myocarditis (e.g., elevated troponin, CPK-MB, or ST2 above the laboratory ULN); or 2.) has evidence of cardiac arrhythmia and/or ST elevation > 1mm, QRS complex changes (Q waves > 0.04 sec and >0.25 of the amplitude of R wave), or symmetric negative T waves AND 3. need for inotropic support. **Definition B**–Participant meets all three criteria: 1.) has an acute illness with discrete onset of signs and symptoms consistent with acute viral myocarditis (e.g., elevated troponin, CPK-MB, or ST2 above the laboratory ULN); 2.) has evidence of cardiac arrhythmia and/or ST elevation > 1mm, QRS complex changes (Q waves > 0.04 sec and >0.25 of the amplitude of R wave), or symmetric negative T waves; and 3. need for inotropic support. **Revised Definition A**–Participant meets criteria 1 or 2, plus criteria 3: 1.) has an acute illness with discrete onset of signs and symptoms consistent with acute viral myocarditis (e.g., elevated troponin, CPK-MB, or ST2 above the laboratory ULN); or 2.) has evidence of cardiac arrhythmia and/or ST elevation > 1mm, QRS complex changes (Q waves > 0.04 sec and >0.25 of the amplitude of R wave) or symmetric negative T waves; and 3.) needs inotropic support AND has evidence of myocardial dysfunction from echocardiogram, that is, reduced left ventricular function, despite adequate filling of left ventricle (normal left ventricle end diastolic diameter. **Newly Revised Definition A**–Participant meets criteria 1 or 2, plus criteria 3: 1.) has an acute illness with discrete onset of signs and symptoms consistent with acute viral myocarditis (e.g., elevated troponin, CPK-MB, or ST2 above the laboratory ULN); or 2.) has evidence of cardiac arrhythmia and/or ST2 elevation > 1mm, QRS complex changes (Q waves > 0.04 sec and >0.25 of the amplitude of R wave), or symmetric negative T waves; and 3 a.) needs inotropic support AND/OR 3 b.) has evidence of myocardial dysfunction from echocardiogram, that is, reduced left ventricular function and has normal volume status (i.e., normal left ventricle end diastolic diameter on ECHO indicating adequate filling of left ventricle).

The software automatically centralized all data for tabulation and a qualitative content analysis was done for open-ended questions and a quantitative analysis for closed-ended questions. Anonymous responses were used to modify the endpoint and operational recommendations in successive rounds until at least 70% agreement, a pre-specified cut-off, of those responding to a given question was reached. If an endpoint definition proposed in round 1 did not reach at least 70% agreement, suggestions from the paired open-ended round 1 question on how to improve the endpoint were used to modify the endpoint. The original endpoint and the modified endpoint(s) would be offered in round 2. This allowed a focus on more uncertain or problematic endpoints in the next round. At the start of each new round, participants were provided a summary of the results from the previous round. Participants were given 2 to 3 weeks to respond in each round. Reminder emails were sent about one week before the close of each round and again 24 hours before the deadline. The Humanities and Social Sciences Ethics Committee at the University of Liège reviewed and approved the study protocol.

## Results

**Fig 1:**
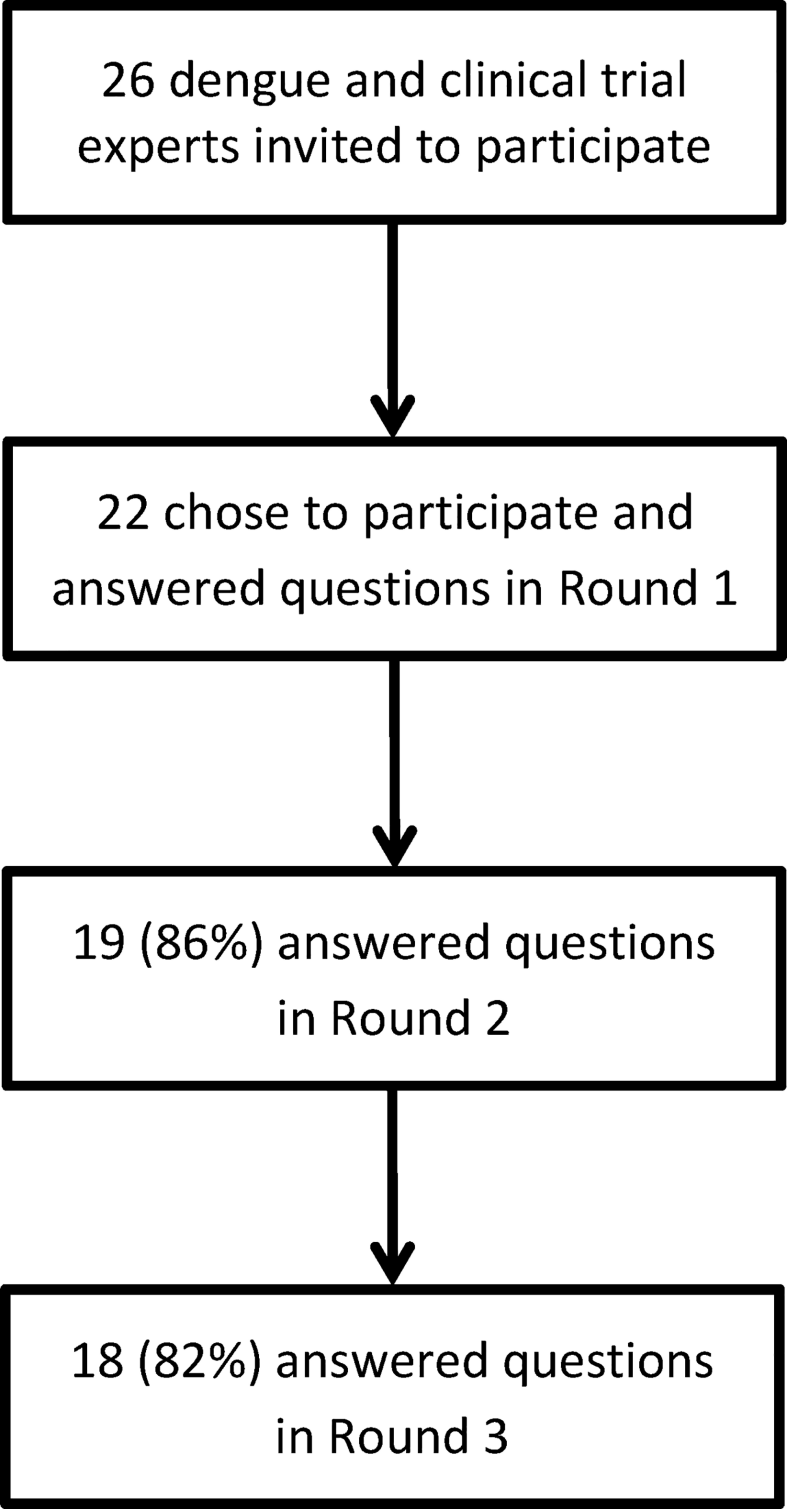
Participants in the Delphi inquiry.

Twenty-six invitations were sent out. There were 22 active respondents (defined as persons who answered at least one question) in the first round with 91% completing all non-conditional questions, 19 active respondents (94% completion rate) in the second round, and 18 active respondents (92% completion rate) in the third round (Fig 1). Professional sector activities were as follows: 27% industry/vaccine developers, 54% academia, 27% public health, 35% clinical sector, 35% governmental sector, and 4% non-governmental organization. Participants reported working in 27% North America, 12% South-East Asia, 8% South America, 8% Central America, 8% Western Pacific, 8% Europe, and 4% Africa. One in four participants (27%) reported working in two or more countries or regions. Most (92%) reported acting in the capacity of a dengue expert.

### Moderate and severe plasma leakage

Most symptomatic dengue patients start to recover around the time of defervescence, usually between days 3–7 of illness. However, in a small proportion of cases, an increase in vascular permeability becomes clinically apparent around this time, marking the onset of the critical phase for complications. The altered permeability results in plasma leakage, evidenced variously by pleural effusions, ascites, hemoconcentration and/or hypoproteinaemia. In severe cases and in the absence of appropriate fluid resuscitation, leakage may compromise the circulating plasma volume so that the patient develops potentially life-threatening hypovolemic shock [18]. Based on a literature review, definitions for moderate and severe plasma leakage were developed and presented to participants (Table 1). To operationalize the endpoints, hemoconcentration was defined as a >15% or >20% change in hematocrit [19, 20].

In round 1, the majority (73%) of participants preferred moderate plasma leakage Definition A, which relies on a hematocrit change of >15%, over Definition B which relies on a hematocrit change of >20% (Table 1 and S1 Table). While 64% selected severe plasma leakage Definition A in round 1 (Table 1 and S1 Table), by round 3 the majority (72%) of participants felt that different hematocrit cut-offs should be used for moderate versus severe plasma leakage and that a >20% cut-off should be used for severe plasma leakage. When asked to select other case-defining factors that should be captured as “evidence of plasma leakage,” which was part of severe plasma leakage Definition A, most selected pleural effusion (79%) and ascites (79%). The majority of participants felt that gallbladder wall thickening on its own was insufficient evidence of plasma leakage (74%) and that hypoproteinemia should not be case-defining (72%). Options were given to refine the endpoint definitions, and most (83% and 78%, respectively) agreed to add two caveats to the definitions: 1.) “pleural effusion, ascites, or cardiac effusion is a new clinical finding and unrelated to another cause”, and 2.) “if a cardiac effusion is detected without concurrent pleural effusion or ascites then another diagnosis should be considered—such as myocarditis”. Lastly, most participants agreed with the proposed definitions of respiratory compromise (83%) and hemodynamic instability (78%) (Table 1).

*Hemoconcentration*. A change in hematocrit from a baseline value is required to define hemoconcentration. The majority (77%) of participants chose baseline hematocrit definitions B or D, which differed only by allowing use of population standards for hematocrit (Table 1 and S1 Table). However, in round 2 no agreement was reached regarding whether population standards can be used when no other value is available, so this was not included in the final definition (Table 2). After two rounds of discussion, most (83%) participants agreed with defining maximum hematocrit as the peak value recorded during the acute illness taken during a period consistent with expected plasma leakage (day 4–8 from fever onset) or within 48 hours of defervescence (S1 Table and Table 2).

**Table 2:** Summary of final clinical endpoint definitions and operational considerations.

Clinical Endpoint Definitions	Operational Considerations
***Moderate plasma leakage*.** Clinical trial participant, with no evidence of hemodynamic instability or respiratory compromise, has evidence of plasma leakage defined by: 1.) >15% change in hematocrit during the illness, and/or 2.) evidence of a new pleural effusion, pericardial effusion, or ascites on ultrasound or x-ray.	• The key clinical feature that differentiates moderate from severe plasma leakage cases is the absence of hemodynamic instability and/or respiratory compromise resulting from plasma leakage (i.e., not the result of bleeding, sepsis or secondary infection). • To determine if a clinical trial participant has evidence of plasma leakage, the following data should be collected: 1.) percent change in hematocrit during the illness (e.g., an increase in hematocrit during critical period from baseline hematocrit); and 2.) presence of a pleural effusion, ascites, and/or cardiac effusion. ○ Effusions should be new and not attributable to another cause or underlying medical condition. If a cardiac effusion is detected without concurrent pleural effusion or ascites, another diagnosis should be considered, such as myocarditis. ○ Gallbladder wall thickening on its own (i.e., without another effusion) is not sufficient evidence of plasma leakage. ○ Hypoalbuminemia or hypoproteinemia on its own is not sufficient evidence of plasma leakage. • Hemoconcentration is defined by the formula: (maximum hematocrit–baseline hematocrit) / baseline hematocrit x 100), with a minimum of three values obtained during the acute illness. ○ Baseline hematocrit is defined as the lowest hematocrit value from among the following: 1.) before plasma leakage typically becomes apparent (first 72 hours of illness) or at study entry, 2.) after plasma leakage stabilizes (day 8 or more, a minimum of 24 hours after stopping IV fluid), or 3.) at a follow-up visit during convalescence (i.e., 14 to 28 days after onset). ○ Maximum hematocrit is defined as the highest hematocrit value recorded during the acute illness taken during the time-period consistent with expected plasma leakage (day 4–8 after fever onset) or within 48 hours of defervescence.
***Severe plasma leakage*.** Clinical trial participant has evidence of hemodynamic instability or respiratory compromise, and evidence of plasma leakage defined by: 1.) >20% change in hematocrit during the illness; and/or 2.) evidence of a new pleural effusion, pericardial effusion, and ascites on ultrasound or x-ray.	• Hemodynamic instability is defined by presence of narrow pulse pressure less than or equal to 20 mmHg, or low systolic blood pressure (SBP) for age, or a drop in SBP >40 during course of the illness (e.g., in adult with chronic hypertension); and two or more signs of shock including: 1.) elevated heart rate for age; 2.) pale, cool skin; 3.) thready, weak pulse; or 4.) capillary refill delayed >2 seconds. • Respiratory compromise is defined by presence of: 1.) an increased respiratory rate for age; 2.) one or more signs of increased work of breathing (e.g., retractions, nasal flaring, accessory muscle use); and 3.) need for additional support to include oxygen supplementation or intubation.
***Moderate bleed*.** Clinical trial participant has bleeding with no evidence of shock or hemodynamic instability, but a local intervention is needed. This includes the following bleeds: • Large skin/injection site bleed needing pressure compress; • Nose/gum bleed needing local intervention (e.g., nasal or gum packing, use of adrenaline); • Gastrointestinal bleeding without shock or hemodynamic instability, or need for blood product but the bleed warrants that a type and crossmatch be done and closer monitoring; • Vaginal bleed with need for hormonal therapy and type and crossmatch to be done; • Any bleed that persists after local measures are taken to stop bleeding (e.g., application of pressure) and bleed requires more intensive monitoring in an ICU or HDU. [Note: there was no need for blood transfusion.]	When collecting data to describe and determine the severity of bleeding among clinical trial participants, investigators should consider the following: • Finding that a type and cross match was ordered does not serve as a reliable indicator of bleeding severity. That is, it does not meet criteria as a local intervention on its own. • Periorbital bleed with periorbital swelling can be a moderate bleed if it requires need for pressure compress (see large skin/injection site bleed needing pressure compress). • “Need for blood transfusion” should be defined as needing whole blood or packed red blood cells. If fresh frozen plasma, platelets or factor concentrates are given without whole blood or packed red blood cells then it is unlikely that the bleed is clinically severe. Prophylactic platelet transfusions or fresh frozen plasma given for clinical laboratory values (i.e., no clinical indication) are not case defining. • The finding of macroscopic hematuria alone is not considered a moderate bleed. May be considered as a moderate bleed if a local intervention is needed.
***Severe bleed*.** Clinical trial participant has any one of the following four types of bleeding listed below: • Bleed into a critical organ (e.g., CNS bleed) • Bleed leading to hemodynamic instability • Bleed resulting in death or permanent disability (e.g., CNS bleed; intraocular bleed) • Bleed that results in need for blood transfusion AND requires more intensive monitoring in an ICU or HDU.	
***Moderate thrombocytopenia*.** Clinical trial participant has a platelet count in the range of 50,000 to 20,000 mm^3^ during the illness, and requires more intensive observation on a regular ward, or transfer to HDU or ICU depending on facilities available.	• One platelet count daily during the critical phase should be adequate to determine if the patient meets clinical endpoint definition for moderate versus severe thrombocytopenia.
***Severe thrombocytopenia*.** Clinical trial participant has a platelet count less than 20,000 mm^3^ during the illness, and requires more intensive observation on a regular ward, or transfer to HDU or ICU depending on facilities available.	
***Moderate liver disease (acute hepatitis)*.** Clinical trial participant meets all 3 criteria: 1.) has an acute illness with discrete onset of signs and symptoms consistent with acute viral hepatitis (e.g., fatigue, abdominal pain, loss of appetite, intermittent nausea, vomiting, dark urine, clay-colored or light stools); 2.) alanine aminotransferase (ALT) greater than 10 times the upper limit of normal consistent with grade 4 toxicity in the FDA 2007 toxicity tables or > = 400 U/L; and 3.) does not meet criteria for acute liver failure (i.e., no mental status changes and international normalized ratio (INR) <1.5).	• Recommend use of acute hepatitis as the moderate liver disease endpoint and acute liver failure as a severe liver disease endpoint. • ALT should be evaluated for all dengue clinical trial participants with an acute febrile illness. • Two or more ALT measurements should be done depending on severity of illness with a third ALT (or more) recommended if the case is severe, the second ALT is elevated, or local clinical practice dictates that more ALT measurements be done.
***Severe liver disease (acute liver failure)*.** Clinical trial participant meets all 3 criteria: 1.) has an acute clinical syndrome consistent with acute hepatitis; 2.) new onset change in mental status or any grade of hepatic encephalopathy that occurs after onset of hepatitis; and 3.) new onset coagulopathy as demonstrated by an international normalized ratio (INR) greater than or equal to 1.5.	
***Moderate neurologic disease*.** Clinical trial participant meets all 3 criteria: 1.) has an abnormal neurologic examination with a Glasgow Coma Score ≥12 but less than 15 for <2 days duration; 2.) neurologic involvement did not result in need for intubation, shunting or intensive care; and 3.) neurologic involvement did not result in death or ongoing sequelae that impairs daily function for more than 48 hours.	• Clinical trial participants with an acute febrile illness and new onset neurologic condition, should have a neurologic examination documented in the record including Glasgow Coma Scale for adults and Blantyre coma score for children. To define if a trial participant meets criteria for moderate or severe neurologic disease, a history of the clinical course, including signs and symptoms, duration of findings, and level of intervention needed or received, should be collected. • Laboratory-confirmed dengue cases should have their neurologic condition classified using evidence-based definitions developed by consensus groups (e.g., the Brighton Collaboration definitions). Other data that may need to be collected to classify the neurologic case include: • Findings from cerebral spinal tap including cerebral spinal fluid (CSF) examination of cells, protein and glucose level, etc. • Results of the CSF PCR for DENV. • Findings from CSF and serum diagnostic assays done to rule out co-infection with other neurotropic pathogens in the area (e.g., Zika virus, West Nile virus, Japanese encephalitis virus, YFV, malaria, etc.). • Findings from brain imaging: CT scan or MRI of brain. • Findings from EMG. Cases with signs and symptoms consistent with Guillain–Barré syndrome or another paralytic condition should have EMG done if available. • Findings from the brain tissue histopathology if autopsy is done. Tissue may be needed to make a definitive diagnosis as per Brighton Collaboration definitions.
***Severe neurologic disease*.** Clinical trial participant has an abnormal neurologic examination with a Glasgow Coma Score <11 in adults or a Pediatric Glasgow Coma Scale <11 or a Blantyre coma score <3 in children; AND subject has: 1.) neurologic involvement resulting in death or ongoing sequelae that impairs daily function, and /or 2.) they received or was thought to require intubation, shunting, or intensive care or high dependency unit level of care if an intensive care unit is not available.	
***Moderate myocarditis*.** Clinical trial participant meets either criteria: 1.) has an acute illness with discrete onset of signs and symptoms consistent with acute viral myocarditis (e.g., elevated troponin, CPK-MB, or ST2 above the laboratory upper limits of normal), or 2.) has evidence of new onset cardiac arrhythmia and/or ST2 elevation > 1mm, QRS complex changes (Q waves > 0.04 sec and >0.25 of the amplitude of R wave), or symmetric negative T waves.	• An electrocardiogram (ECG) should be conducted on clinical trial participants with an acute febrile illness who develop clinical cardiac signs and symptoms consistent with myocarditis
***Severe myocarditis*.** Clinical trial participant meets either criteria 1 or 2, plus criteria 3: 1.) has an acute illness with discrete onset of signs and symptoms consistent with acute viral myocarditis (e.g., elevated troponin, CPK-MB, or ST2 above the laboratory ULN); or 2.) has evidence of new onset cardiac arrhythmia and/or ST elevation > 1mm, QRS complex changes (Q waves > 0.04 sec and >0.25 of the amplitude of R wave), or symmetric negative T waves; AND 3.) has need for inotropic support, and/or has evidence of myocardial dysfunction from echocardiogram, that is, reduced left ventricular function, despite adequate filling of left ventricle (normal left ventricle end diastolic diameter) and adequate volume status.	

### Moderate and severe bleeding

Bleeding is a common manifestation in dengue patients, with cutaneous bleeding, epistaxis or gum bleeding being more common than gastrointestinal or vaginal hemorrhage [6, 20–27]. The frequency and severity of clinically significant bleeding is thought to vary by patient age and disease severity such that adult cases and those with dengue shock syndrome are more likely to have severe spontaneous bleeding than pediatric cases and those without shock [6, 20, 26–29]. Several grading systems for bleeding exist [30, 31] and were used to derive criteria for moderate and severe bleeding that were presented to participants (Table 1). Bleeding that requires a local intervention but does not result in shock or hemodynamic instability were the two key criteria that define moderate bleeding. Proposed severe bleeding included any bleeding that involves a critical organ; leads to hemodynamic instability; results in death or permanent disability; or requires a red blood cell transfusion and more intensive monitoring in an intensive care unit or high dependency unit.

In round 1, more than 70% of participants agreed that Definitions A-D and F represented moderately severe bleeding, while Definitions E (macroscopic hematuria) and G (eye bleeding that does not affect vision) did not get this level of agreement (Table 1 and S2 Table). In subsequent rounds, the majority (74%) agreed that the finding that a blood type and cross match was ordered should not be an indicator of severity on its own and moderate bleeding definitions A and F were modified accordingly (Table 2). In round 1, most (>90%) selected Definitions A through D as severe grade bleeding, while 64% preferred Definition E for severe bleeding (Table 1 and S2 Table). In round 2, most (79%) participants agreed that if there is no need for blood transfusion, the bleeding described in Definition E should define moderate bleeding. In addition, 79% agreed with the statement that “need for blood transfusion” be defined as meaning need for whole blood or packed red blood cells.

### Moderate and severe thrombocytopenia

Thrombocytopenia is a common finding in patients with dengue. Although pathogenesis is incompletely understood, it likely includes direct and indirect viral-mediated mechanisms [32, 33]. Traditionally, thrombocytopenia has been used as a criterion for dengue disease severity [34]. However, as a predictive marker for bleeding, the speed of the platelet count decrease may be more important than the absolute number. Based on a review of the literature and discussions, definitions for moderate and severe thrombocytopenia were developed for participants to consider (Table 1).

In round 1, a majority (68%) of participants chose moderate thrombocytopenia Definitions B or C, which specified a 50,000 to 20,000 mm^3^ platelet count range as case defining (Table 1 and S2 Table). While participants were more divided over the four severe thrombocytopenia endpoints, 55% of participants chose severe thrombocytopenia Definitions B or C which specified a <20,000 mm^3^ platelet count cut-off. Participants remained divided through round 3 on whether moderate and severe thrombocytopenia Definition B or C was best for use in clinical trials. The difference between these definitions is that both moderate and severe thrombocytopenia Definition B specify the need to detect a decrease in the platelet count within the specified range in a 24-hour period, while both Definition C specify that one platelet count within the range is case defining (Tables 1 and 2). The most common reason for support of Definition B was that a single value could be a spurious measurement, whereas a fall within a 24-hour period is more meaningful. In contrast, those who chose Definition C felt it was easier to operationalize because only one platelet measurement is needed each day and any value within the range would be considered case defining. Last, 72% felt that one platelet count measurement done daily during the critical phase is adequate to determine if a case-patient had severe or moderate thrombocytopenia. Operationally, this is more consistent with Definition C.

### Moderate and severe liver disease

Liver involvement in dengue patients is relatively common and serum aminotransferases are elevated in most hospitalized dengue patients, with higher levels observed among severe dengue cases [35–42]. Prospective studies have found that acute hepatitis occurs in 2–6% of dengue inpatients when defined by an ALT >10 times the upper limit of normal [37, 43]. Acute liver failure (ALF) is also known to occur among confirmed dengue patients [38–42], even among patients without shock, but ALF is uncommon [39]. A review of the literature found several definitions for ALF [44], however, most included acute liver dysfunction with change in mental status and new onset coagulopathy defined by an international normalization ratio (INR) ≥1.5 as case-defining criteria [45–48]. There were fewer definitions for acute viral hepatitis [35–40, 43], and distinguishing criteria included use of an ALT cut-off alone versus AST and/or ALT, and presence of jaundice. The working group recommended the use of an ALT cut-off because of its greater specificity for liver involvement than AST, and the use of an ALT >10 times the upper limits of normal cut-off, which corresponds to Grade 4 FDA toxicity table level (Table 1). Presence of jaundice or elevated bilirubin were not recommended because of difficulties in evaluating jaundice in populations with dark skin pigmentation.

In round 1, most (77%) participants selected Definition A for acute hepatitis and Definition A for ALF (Table 1 and S3 Table). In round 2, 84% agreed that ALF should be the severe liver disease endpoint while acute hepatitis should be the moderately severe liver disease endpoint, and most (84%) participants felt that ALT should be evaluated for all trial participants with an acute febrile illness. In round 3, 78% agreed that, “at least two ALT levels should be done depending on the severity of illness with a third ALT (or more) recommended if a case is severe; the second ALT is elevated; or local clinical practice indicates more ALT measurements be done”. Last, participants were asked whether there should be a recommendation that trial participants with an AFI have an INR measured, and if so, the timing of the measurement. Many (61%) participants disagreed with making the recommendation.

### Moderate and severe neurologic disease

Reports of neurologic disease in dengue patients are rare despite a high burden of dengue. However, neurologic disease has been described in laboratory-confirmed dengue cases involving all DENV types, all age groups, and in all parts of the world [49–51]. Dengue encephalitis [52–64] and encephalopathy [63–66] are most commonly described followed by Guillain-Barré Syndrome and aseptic meningitis; however, other neurologic conditions have been reported [64, 65, 67–69]. The incidence of neurologic disease among dengue patients is difficult to determine because case definitions, study population and methods vary among studies, but estimates have ranged from 0.5–20% [49, 64, 68, 70]. Based on a literature review, it was proposed that laboratory-confirmed dengue cases in clinical trials with an abnormal neurologic examination be defined as moderate or severely affected and that these cases then be classified using established case definitions (Table 1). Further, given the past difficulties in attribution, it was proposed that alternative etiologies, including concurrent metabolic abnormalities and co-infections with other neurotropic flaviviruses and pathogens be assessed.

In round 1, 68% of the participants chose moderate neurologic disease Definition C; however, some participants commented that a specific Glasgow Coma Score (GCS) should be added to the definition to make it more measurable instead of including it as a footnote (Table 1 and S3 Table). In round 2, the majority (74%) agreed that a specific GCS be added to moderate neurologic disease Definition C (S3 Table and Table 2). In round 1, the majority (73%) of participants chose severe neurologic disease Definition C, and 63% agreed to add GCS to the severe neurologic disease definition in round 2. Reasons cited for not adding GCS included inconsistent use of the score depending on where and how participants are treated, and poor interrater reliability. Some participants felt that capturing GCS was inconsequential compared with need for neurologic intervention and the duration of neurologic impairment. However, in round 3, most (83%) agreed to a modified severe neurologic disease Definition C in which use of a high dependency unit was added (Table 1). Last, when asked questions about how to operationalize the neurologic disease endpoints, a majority (68%) participants recommended that evidence-based definitions such as those developed by the Brighton Collaboration be used to further classify identified neurologic cases. When asked to select data to be collected for trial participants with neurologic disease (Tables 1 and 2), the majority (>70%) agreed that items 1 and 2 should be required. False positive rates of PCR, difficulty in collecting cerebral spinal fluid (CSF) on dengue patient with bleeding tendencies, lack of EMG at some facilities, and lack of availability of post-mortem tissue were cited by participants as reasons items 3 through 8 should be optional. However, these items could be collected if readily feasible at the participating trial site or if clinical presentation warrants further investigation (e.g., electromyography in suspected Guillain-Barré Syndrome case).

### Moderate and severe myocarditis

Reduced cardiac output and myocarditis have been reported in dengue-infected patients although the incidence is unknown, and the severity of cardiac involvement has not been well-characterized [71–76]. A direct myodepressive effect of dengue virus has been difficult to determine because cardiac function is preload-dependent and dengue patients can have reduced intravascular volume secondary to a vascular leakage syndrome [72, 77]. Electrocardiogram (ECG) abnormalities, bradycardia, and conduction abnormalities have been described, however, cardiac enzymes are not commonly elevated in dengue, even in cases with suspected myocarditis [72, 75, 77, 78]. Most cardiac involvement in dengue patients appears to be transient as the ejection fraction and conduction abnormalities return to normal during convalescence [75, 78, 79]. Because of the difficulty in discerning a true viral-induced myodepressive effect from that due to reduced intravascular volume, myocarditis was chosen as the clinically-relevant cardiac endpoint in clinical trial participants with laboratory-confirmed dengue. Based on a review of the literature, proposed definitions for moderate and severe myocarditis were developed for participants to consider (Table 1).

In round 1, the more sensitive endpoint definitions were selected more often with 55% of participants choosing Definition A for moderate myocarditis and a similar proportion (59%) choosing Definition A for severe myocarditis (Table 1 and S3 Table). In round 2, the majority (74%) agreed that we should specify that the arrhythmia in the definition should be a “new onset” arrhythmia, and many (58%) of the participants felt that we should recommend that ECGs be done only for those with clinical findings consistent with cardiac involvement. In round 2, 68% of participants agreed that the phrase: “has evidence of myocardial dysfunction on the echocardiogram, that is, reduced left ventricular function, despite adequate filling of left ventricle (normal left ventricle end diastolic diameter) and adequate volume status” be added to the definition. Less than half (47%) of participants preferred that the caveat, “need for inotropic support AND has evidence of myocardial dysfunction from echocardiogram” be added to criteria 3 in severe myocarditis Definition A. In the end, participants were divided on whether the revised severe myocarditis Definition A (39%) or the newly revised severe myocarditis Definition A (39%) should be used in clinical trials. However, in round 3, participants were given a scenario and asked how they would classify a trial participant with laboratory-confirmed dengue who has ST elevation on an ECG, elevated cardiac enzymes, and need for inotropic support who did not have an ECHO done. A majority (72%) of participants stated that they would classify this case as having severe myocarditis, which is consistent with the newly revised Definition A (Tables 1 and 2). That is, a case would qualify as severe if they receive inotropic support, and/or they have evidence of myocardial dysfunction from echocardiogram.

## Discussion

We set out to define separate endpoints for plasma leakage, bleeding and organ impairment, to more precisely characterize clinical phenotypes among research study participants with laboratory-confirmed dengue. There was at least 70% agreement on eight of the 12 clinical endpoint definitions that we addressed, including moderate and severe plasma leakage, moderate and severe bleeding, moderate and severe liver disease, and moderate and severe neurologic disease. Not surprisingly, there was less agreement among participants on the definitions for myocarditis, an endpoint which is an uncommon manifestation of dengue. In addition, for moderate and severe thrombocytopenia, although consensus was reached on certain parameters, some issues, primarily practical, remain to be addressed. Although further work is needed to finalize these remaining endpoints, we feel the proposed definitions as described in Table 2 should now be made available to interested groups to begin the process of evaluation.

We envisage that achieving any one severe or moderate endpoint during a laboratory-confirmed dengue illness would be sufficient to designate the case as severe or moderate, respectively. However, we suggest that the research community might benefit if data on all endpoints were to be presented in reports, thereby describing the clinical outcomes more fully and allowing detailed comparisons between studies. Importantly, researchers should understand that these endpoints are intended to represent the level of severity experienced by a study participant over the course of their laboratory-confirmed dengue illness. For example, to assess a participant for moderate or severe plasma leakage, the investigator should evaluate hematocrit at different times during the illness to evaluate hemoconcentration.

With respect to thrombocytopenia, although we attempted to characterize severe and moderate thrombocytopenia, it was not our intention for a case with severe thrombocytopenia alone, in the absence of any other severe criterion, to be classified as a severe dengue case. Although historically thrombocytopenia was part of the definition of dengue hemorrhagic fever and considered to be an indicator of disease severity, in the WHO 2009 classification, thrombocytopenia alone is not included as part of the definition for severe dengue. Agreement on moderate and severe thrombocytopenia endpoint definitions did not reach the level of agreement attained by plasma leakage, bleeding, and liver and neurologic disease endpoints. However, a clear majority (68%) of participants agreed on a 50,000–20,000 mm^3^ platelet count range for moderate thrombocytopenia, and more than half (58%) agree to a <20,000 mm^3^ platelet count cut-off for severe thrombocytopenia in round 1. By round 3, the only remaining issue which divided respondents was whether one platelet count or a decreasing trend within the cut-off range was case-defining. However, in the end the majority felt that one platelet count measurement done daily during the critical phase is adequate to determine if a case-patient had severe or moderate thrombocytopenia which is, operationally, more consistent with Definition C (i.e., needing one platelet count within a cut-off range).

Over 70% agreement was reached early on most types of moderate and severe bleeding. Eye bleeding not affecting vision was dropped from the list of types of moderate bleeding; however, eye bleeding resulting in permanent disability will be captured under Definition C for severe bleeding. Macroscopic hematuria nearly had reached 70% agreement; however, it was rejected by some because no intervention was mentioned in the definition and all the other types of moderate bleeding specified a need for an intervention. Similarly, Definition E for severe bleeding was thought not to be a severe because there was no need to give whole blood or packed red blood cells, and most participants agreed that it be included as moderate bleeding. Lastly, the majority of participants agreed that, “need for blood transfusion” meant need for whole blood or packed red blood cells. This is important as studies have found that prophylactic platelet transfusions are not uncommonly given to dengue patients [80–82]. In addition, the sentiment among many participants was if fresh frozen plasma, platelets or factor concentrates were given without whole blood or packed red blood cells, then it was unlikely that the bleeding was clinically severe.

Patients with dengue may have significant organ involvement, with the most commonly affected organ being the liver. Most participants agreed early on to definitions for acute hepatitis and acute liver failure without changes to the proposed definitions. However, it was more difficult to reach agreement on operational items, including timing and number of ALT measurements and need for INR. In the end, most recommended that two or more ALT levels be obtained during the clinical course.

While neurologic disease is thought to be infrequent, it is part of the current WHO classification for severe dengue and an outcome that may be associated with significant morbidity and mortality. One of the issues with determining the incidence of neurologic disease among laboratory-confirmed dengue cases has been the inconsistent use of case definitions and shortcomings with regards to data collection and methodology [49–51]. We therefore sought to propose a sensitive clinical endpoint definition and systematic collection of data that would enable the description of a broad spectrum of moderate and severe neurologic outcomes among clinical trial participants with laboratory-confirmed dengue. Once a case is identified with moderate or severe neurologic disease, cases can be classified using evidence-based definitions such as those developed by the Brighton Collaboration [83]. We reached agreement on endpoint definitions for moderate and severe neurologic disease. However, there was less agreement on recommendations for data to be collected for neurologic disease cases. Many of the proposed items for data collection depend on feasibility and clinical presentation.

While the process enabled us to reach agreement on most endpoints from a geographically dispersed group of experts, there were some challenges. First, participants were extremely busy individuals and even with frequent reminders, participation decreased by 18% between the first and third round. We tried to minimize drop-out by using an easily accessible online platform to query participants that allows direct communication with participants for technical support. We also sent email reminders between rounds. Second, early on it became clear that there were a few dominant individuals in the working group. By using a Delphi methodology-based query, participants do not interact with each other thus allowing all expert opinion to be heard. In addition, the online platform allowed for more in-depth responses to the open-ended questions since participants were able to save and revisit their answers during the round. In this way, we were able to get more agreement than would have been possible in additional face-to-face meetings. Third, while Delphi methodology-based queries have the potential to create a bottleneck towards convergence of opinions [84], we felt we were able to prevent this from happening by using a combination of close and open-ended questions to capture opinions. Last, operational considerations were discussed at length throughout the process, and in the end, our endpoints can be used in all settings. However, further classification of moderate and severe neurologic cases may be a challenge in settings with limited resources (e.g., Guillain–Barré case).

Outlook: With over 70% agreement on most clinical endpoint definitions, a group of dengue experts is working to validate the endpoints using several large existing prospective data sets. Specifically, they will evaluate endpoint accuracy in identifying moderate and severe disease, endpoint reproducibility in diverse clinical settings, and ease of use. In addition, they will assess how many dengue cases could not be included because necessary components of the endpoint definition, such as repeat clinical laboratory tests or clinical assessments, were not performed. For example, multiple data points may not be available for non-hospitalized dengue cases identified in community-based clinical studies. Such cases are likely to be non-severe; however, there is currently insufficient data to rule out moderate severity. Last, more work is needed to finalize the myocarditis endpoints, and evaluate the utility of one versus two or more platelet count measurements within a 24-hour period to identify cases with moderate and severe thrombocytopenia. The proposed clinical endpoints can be used to harmonize data collection and improve comparability between dengue clinical trials.

## Supporting information

S1 Table Responses to questions about plasma leakage by round of inquiry. (DOCX)

S2 Table Responses to questions about bleeding and thrombocytopenia by round of inquiry. (DOCX)

S3 Table Responses to questions about liver, neurologic and cardiac disease by round of inquiry. (DOCX)
