# Supplementary material for: Development of standard clinical endpoints for use in dengue interventional trials
Source: PLoS Negl Trop Dis. 2018 Oct 4;12(10):e0006497. doi: 10.1371/journal.pntd.0006497 (PMC6171842; doi:10.1371/journal.pntd.0006497)
Supplement: S1 Table — (DOCX) [file pntd.0006497.s001.docx]

| **Supplemental Table 1. Responses to questions about plasma leakage by round of inquiry** | | | | | | | | | | | | | |
| --- | --- | --- | --- | --- | --- | --- | --- | --- | --- | --- | --- | --- | --- |
| **Questions about the plasma leakage clinical endpoint definitions and operation items**^*^ | Question Number | **Round 1 (n=22)^**^** | | | | **Round 2 (n=19)^**^** | | | | **Round 3 (n=18) ^**^** | | | |
|  |  | **Agree** | | **Disagree** | | **Agree** | | **Disagree** | | **Agree** | | **Disagree** | |
|  |  | **No.** | **(%)** | **No.** | **(%)** | **No.** | **(%)** | **No.** | **(%)** | **No.** | **(%)** | **No.** | **(%)** |
| Prefer moderate plasma leakage Definition A vs. Definition B | 1.5.7 | 16 | (73) | 3 | (14) |  |  |  |  |  |  |  |  |
| Prefer severe plasma leakage Definition A vs. Definition B | 1.5.9 | 14 | (64) | 6 | (27) |  |  |  |  |  |  |  |  |
| Felt % increase in hematocrit part of moderate definition | 2.9.1 |  |  |  |  | 14 | (74) | 2 | (11) |  |  |  |  |
| Prefer >15% cut-off versus >20% for moderate plasma leakage | 2.9.2 |  |  |  |  | 11 | (58) | 4 | (21) |  |  |  |  |
| Severe leakage differs by shock or respiratory compromise | 2.9.4 |  |  |  |  | 12 | (63) | 4 | (21) |  |  |  |  |
| Felt should be different HCT cut-offs for moderate vs. severe | 3.10.1 |  |  |  |  |  |  |  |  | 13 | (72) | 3 | (17) |
| Prefer hematocrit cut-off of >20% for severe plasma leakage | 3.10.2 |  |  |  |  |  |  |  |  | 13 | (72) | 0 | (0) |
| Felt pleural effusion (PE) is evidence of plasma leakage | 2.8.1 |  |  |  |  | 15 | (79) | 1 | (5) |  |  |  |  |
| Felt ascites is evidence of plasma leakage | 2.8.1 |  |  |  |  | 15 | (79) | 1 | (5) |  |  |  |  |
| Felt change in hematocrit is evidence of plasma leakage | 2.8.1 |  |  |  |  | 13 | (68) | 3 | (16) |  |  |  |  |
| Felt cardiac effusion is evidence of plasma leakage | 2.8.1 |  |  |  |  | 12 | (63) | 4 | (21) |  |  |  |  |
| Felt gallbladder wall thickening is evidence of plasma leakage | 2.8.1 |  |  |  |  | 4 | (21) | 12 | (63) |  |  |  |  |
| Felt gallbladder wall thickening alone not sufficient evidence | 2.8.3 |  |  |  |  | 14 | (74) | 2 | (11) |  |  |  |  |
| Add hypoproteinemia as case-defining plasma leakage criteria | 3.9.3 |  |  |  |  |  |  |  |  | 3 | (16) | 13 | (72) |
| Limit “evidence of fluid” to PE, cardiac effusions and ascites | 2.8.4 |  |  |  |  | 13 | (68) | 2 | (11) |  |  |  |  |
| Felt no need to quantify effusion | 2.8.5 |  |  |  |  | 12 | (63) | 4 | (21) |  |  |  |  |
| Felt should not recommend that pleural effusion be quantified | 3.9.5 |  |  |  |  |  |  |  |  | 12 | (67) | 4 | (22) |
| Felt should not recommend that ascites be quantified | 3.9.7 |  |  |  |  |  |  |  |  | 14 | (78) | 2 | (11) |
| Add caveat: effusions are new and unrelated to another cause | 3.9.1 |  |  |  |  |  |  |  |  | 15 | (83) | 1 | (6) |
| Add caveat: if cardiac effusions alone evaluate for myocarditis | 3.9.2 |  |  |  |  |  |  |  |  | 14 | (78) | 2 | (11) |
| Agree with proposed definition of hemodynamic instability | 3.10.7 |  |  |  |  |  |  |  |  | 14 | (78) | 2 | (11) |
| Agree with proposed definition of respiratory compromise | 3.10.10 |  |  |  |  |  |  |  |  | 15 | (83) | 1 | (6) |
| Felt severe plasma leakage should include hemodynamic instability and need for supportive care beyond initial efforts | 3.10.9 |  |  |  |  |  |  |  |  | 11 | (61) | 5 | (28) |
| Prefer hemoconcentration Definition A | 1.5.5 | 0 | (0) | 19 | (0) |  |  |  |  |  |  |  |  |
| Prefer hemoconcentration Definition B | 1.5.5 | 10 | (45) | 9 | (41) |  |  |  |  |  |  |  |  |
| Prefer hemoconcentration Definition C | 1.5.5 | 9 | (41) | 10 | (45) |  |  |  |  |  |  |  |  |
| Prefer baseline hematocrit Definition A | 1.5.3 | 0 | (0) | 19 | (86) |  |  |  |  |  |  |  |  |
| Prefer baseline hematocrit Definition B | 1.5.3 | 10 | (45) | 9 | (41) |  |  |  |  |  |  |  |  |
| Prefer baseline hematocrit Definition C | 1.5.3 | 2 | (9) | 17 | (77) |  |  |  |  |  |  |  |  |
| Prefer baseline hematocrit Definition D | 1.5.3 | 7 | (32) | 12 | (55) |  |  |  |  |  |  |  |  |
| Felt population standard could be used for baseline HCT | 2.10.3 |  |  |  |  | 10 | (53) | 6 | (32) |  |  |  |  |
| Prefer maximum hematocrit Definition A | 1.5.1 | 5 | (23) | 14 | (64) |  |  |  |  |  |  |  |  |
| Prefer maximum hematocrit Definition B | 1.5.1 | 6 | (27) | 13 | (59) |  |  |  |  |  |  |  |  |
| Prefer maximum hematocrit Definition C | 1.5.1 | 4 | (18) | 15 | (68) |  |  |  |  |  |  |  |  |
| Prefer maximum hematocrit Definition D | 1.5.1 | 4 | (18) | 15 | (68) |  |  |  |  |  |  |  |  |
| Need to define maximum hematocrit for hemoconcentration | 3.10.4 |  |  |  |  |  |  |  |  | 13 | (72) | 3 | (6) |
| Like new proposed definition of maximum hematocrit | 3.10.5 |  |  |  |  |  |  |  |  | 15 | (83) | 0 | (0) |

* Question numbers consist of 3 integers separated by a period. The first integer refers to the round of inquiry; the second refers to a specific topic area, and the third is a subgroup of the second.

**Note: the total number of participants who agreed and disagreed to a specific question may not equal the column total of all active participants for that round because of non-responders, that is, participants were not obliged to respond to a specific question to proceed to the next question.
