# Supplementary material for: Development of standard clinical endpoints for use in dengue interventional trials
Source: PLoS Negl Trop Dis. 2018 Oct 4;12(10):e0006497. doi: 10.1371/journal.pntd.0006497 (PMC6171842; doi:10.1371/journal.pntd.0006497)
Supplement: S2 Table — (DOCX) [file pntd.0006497.s002.docx]

**Supplemental Table 2. Responses to questions about bleeding and thrombocytopenia by round of the inquiry**

| **Questions about the bleeding and thrombocytopenia clinical endpoint definitions and operational items**^*^ | Question Number | **Round 1 (n=22)^**^** | | | | **Round 2 (n=19)^**^** | | | | **Round 3 (n=18)^**^** | | | |
| --- | --- | --- | --- | --- | --- | --- | --- | --- | --- | --- | --- | --- | --- |
|  |  | **Agree** | | **Disagree** | | **Agree** | | **Disagree** | | **Agree** | | **Disagree** | |
|  |  | **No.** | **(%)** | **No.** | **(%)** | **No.** | **(%)** | **No.** | **(%)** | **No.** | **(%)** | **No.** | **(%)** |
| Moderate and Severe Bleeding |  |  |  |  |  |  |  |  |  |  |  |  |  |
| Felt Definition A describes moderate bleeding | 1.1.3 | 17 | (77) | 4 | (19) |  |  |  |  |  |  |  |  |
| Felt Definition B describes moderate bleeding | 1.1.3 | 19 | (86) | 2 | (10) |  |  |  |  |  |  |  |  |
| Felt Definition C describes moderate bleeding | 1.1.3 | 16 | (73) | 5 | (24) |  |  |  |  |  |  |  |  |
| Felt Definition D describes moderate bleeding | 1.1.3 | 17 | (77) | 4 | (19) |  |  |  |  |  |  |  |  |
| Felt Definition E describes moderate bleeding | 1.1.3 | 15 | (68) | 6 | (29) |  |  |  |  |  |  |  |  |
| Felt Definition F describes moderate bleeding | 1.1.3 | 17 | (77) | 4 | (19) |  |  |  |  |  |  |  |  |
| Felt Definition G describes moderate bleeding | 1.1.3 | 10 | (45) | 11 | (52) |  |  |  |  |  |  |  |  |
| Felt no eye bleed would meet criteria for moderate bleeding | 2.5.4 |  |  |  |  | 11 | (58) | 6 | (32) |  |  |  |  |
| Felt type & cross match alone is not indicator of severity | 2.5.5 |  |  |  |  | 14 | (74) | 3 | (16) |  |  |  |  |
| Change Definition A so type & cross not sole intervention | 2.5.6 |  |  |  |  | 15 | (79) | 2 | (11) |  |  |  |  |
| Change Definition F so type & cross not sole intervention | 2.5.7 |  |  |  |  | 13 | (68) | 4 | (21) |  |  |  |  |
| Felt Definition A describes severe bleeding | 1.1.1 | 20 | (91) | 2 | (9) |  |  |  |  |  |  |  |  |
| Felt Definition B describes severe bleeding | 1.1.1 | 21 | (95) | 1 | (5) |  |  |  |  |  |  |  |  |
| Felt Definition C describes severe bleeding | 1.1.1 | 21 | (95) | 1 | (5) |  |  |  |  |  |  |  |  |
| Felt Definition D describes severe bleeding | 1.1.1 | 21 | (95) | 1 | (5) |  |  |  |  |  |  |  |  |
| Felt Definition E describes severe bleeding | 1.1.1 | 14 | (64) | 7 | (32) |  |  |  |  |  |  |  |  |
| Felt Definition E is moderate bleeding as no need for blood | 2.5.1 |  |  |  |  | 15 | (79) | 2 | (11) |  |  |  |  |
| Felt “need for blood” refers to whole blood and PRBCs | 2.5.3 |  |  |  |  | 15 | (79) | 2 | (11) |  |  |  |  |
| Moderate and Severe Thrombocytopenia |  |  |  |  |  |  |  |  |  |  |  |  |  |
| Prefer moderate thrombocytopenia Definition A | 1.1.7 | 2 | (9) | 15 | (68) |  |  |  |  |  |  |  |  |
| Prefer moderate thrombocytopenia Definition B | 1.1.7 | 8 | (36) | 9 | (41) |  |  |  |  |  |  |  |  |
| Prefer moderate thrombocytopenia Definition C | 1.1.7 | 7 | (32) | 10 | (45) |  |  |  |  |  |  |  |  |
| Should not add presence of bleeding to moderate definition | 2.6.3 |  |  |  |  | 15 | (79) | 2 | (11) |  |  |  |  |
| Prefer moderate thrombocytopenia Definition B vs. C | 3.8.1 |  |  |  |  |  |  |  |  | 10 | (56) | 6 | (33) |
| Prefer severe thrombocytopenia Definition A | 1.1.5 | 3 | (14) | 17 | (77) |  |  |  |  |  |  |  |  |
| Prefer severe thrombocytopenia Definition B | 1.1.5 | 5 | (23) | 15 | (68) |  |  |  |  |  |  |  |  |
| Prefer severe thrombocytopenia Definition C | 1.1.5 | 7 | (32) | 13 | (59) |  |  |  |  |  |  |  |  |
| Prefer severe thrombocytopenia Definition D | 1.1.5 | 5 | (23) | 15 | (68) |  |  |  |  |  |  |  |  |
| Prefer severe thrombocytopenia Definition B vs. C | 2.5.8 |  |  |  |  | 10 | (53) | 7 | (37) |  |  |  |  |
| Prefer severe thrombocytopenia Definition B vs. C | 3.7.1 |  |  |  |  |  |  |  |  | 8 | (44) | 8 | (44) |
| Specify need for intensive observation in severe definition | 2.6.1 |  |  |  |  | 11 | (58) | 6 | (32) |  |  |  |  |
| Should have at least 1 platelet count per day in critical phase | 3.7.3 |  |  |  |  |  |  |  |  | 13 | (72) | 3 | (17) |

* Question numbers consist of 3 integers separated by a period. The first integer refers to the round of inquiry; the second refers to a specific topic area, and the third is a subgroup of the second.

**Note: the total number of participants who agreed and disagreed to a specific question may not equal the column total of all active participants for that round because of non-responders, that is, participants were not obliged to respond to a specific question to proceed to the next question.
