# Supplementary material for: Development of standard clinical endpoints for use in dengue interventional trials
Source: PLoS Negl Trop Dis. 2018 Oct 4;12(10):e0006497. doi: 10.1371/journal.pntd.0006497 (PMC6171842; doi:10.1371/journal.pntd.0006497)
Supplement: S3 Table — (DOCX) [file pntd.0006497.s003.docx]

**Supplemental Table 3. Responses to questions about liver, neurologic and cardiac disease by round of the inquiry**

| **Questions about the organ involvement clinical endpoint definitions and operational items**^*^ | Question Number | **Round 1 (n=22)**^**^ | | | | **Round 2 (n=19)** ^**^ | | | | **Round 3 (n=18)** ^**^ | | | |
| --- | --- | --- | --- | --- | --- | --- | --- | --- | --- | --- | --- | --- | --- |
|  |  | **Agree** | | **Disagree** | | **Agree** | | **Disagree** | | **Agree** | | **Disagree** | |
|  |  | **No.** | **(%)** | **No.** | **(%)** | **No.** | **(%)** | **No.** | **(%)** | **No.** | **(%)** | **No.** | **(%)** |
| Moderate and Severe Liver Disease |  |  |  |  |  |  |  |  |  |  |  |  |  |
| Prefer acute hepatitis Definition A vs. B | 1.3.1 | 17 | (77) | 3 | (14) |  |  |  |  |  |  |  |  |
| Prefer acute liver failure (ALF) Definition A vs. B | 1.3.3 | 17 | (77) | 4 | (18) |  |  |  |  |  |  |  |  |
| Recommend acute hepatitis as moderate endpoint, ALF as severe | 2.2.1 |  |  |  |  | 16 | (84) | 1 | (5) |  |  |  |  |
| Should check ALT for dengue trial participants with febrile illness | 2.1.1 |  |  |  |  | 16 | (84) | 2 | (11) |  |  |  |  |
| Felt that one ALT measurement is insufficient | 2.1.3 |  |  |  |  | 11 | (58) | 7 | (37) |  |  |  |  |
| Agree that ≥ 2 ALT needed and more depending on severity | 3.2.7 |  |  |  |  |  |  |  |  | 14 | (78) | 3 | (17) |
| Should specify number of signs/symptoms of hepatitis needed | 3.1.1 |  |  |  |  |  |  |  |  | 5 | (28) | 13 | (72) |
| Should evaluate INR on all dengue trial participants with an AFI | 3.3.1 |  |  |  |  |  |  |  |  | 6 | (32) | 11 | (61) |
| Moderate and Severe Neurologic Disease |  |  |  |  |  |  |  |  |  |  |  |  |  |
| Prefer moderate neurologic disease Definition A | 1.2.3 | 2 | (9) | 17 | (77) |  |  |  |  |  |  |  |  |
| Prefer moderate neurologic disease Definition B | 1.2.3 | 2 | (9) | 17 | (77) |  |  |  |  |  |  |  |  |
| Prefer moderate neurologic disease Definition C | 1.2.3 | 15 | (68) | 4 | (18) |  |  |  |  |  |  |  |  |
| Should add GCS to moderate neurologic disease Definition C | 2.7.1 |  |  |  |  | 14 | (74) | 3 | (16) |  |  |  |  |
| Selected Brighton Collaboration definitions to classify cases | 2.7.3 |  |  |  |  | 13 | (68) | 4 | (21) |  |  |  |  |
| Prefer severe neurologic disease Definition A | 1.2.1 | 2 | (9) | 19 | (86) |  |  |  |  |  |  |  |  |
| Prefer severe neurologic disease Definition B | 1.2.1 | 3 | (14) | 18 | (82) |  |  |  |  |  |  |  |  |
| Prefer severe neurologic disease Definition C | 1.2.1 | 16 | (73) | 5 | (23) |  |  |  |  |  |  |  |  |
| Should add GCS to severe neurologic disease Definition C | 2.6.4 |  |  |  |  | 12 | (63) | 5 | (26) |  |  |  |  |
| Should add HDU to severe neurologic disease Definition C | 2.6.5 |  |  |  |  | 15 | (79) | 2 | (11) |  |  |  |  |
| Prefer modified Definition C for severe neurologic disease | 3.5.1 |  |  |  |  |  |  |  |  | 15 | (83) | 1 | (6) |
| Should recommend data be collected as described Item 1 | 2.7.5 |  |  |  |  | 15 | (79) | 0 | (0) |  |  |  |  |
| Should recommend data be collected as described Item 2 | 2.7.5 |  |  |  |  | 14 | (74) | 1 | (5) |  |  |  |  |
| Should recommend data be collected as described Item 3 | 2.7.5 |  |  |  |  | 12 | (63) | 3 | (16) |  |  |  |  |
| Should recommend data be collected as described Item 4 | 2.7.5 |  |  |  |  | 10 | (53) | 5 | (26) |  |  |  |  |
| Should recommend data be collected as described Item 5 | 2.7.5 |  |  |  |  | 12 | (63) | 3 | (16) |  |  |  |  |
| Should recommend data be collected as described Item 6 | 2.7.5 |  |  |  |  | 11 | (58) | 4 | (21) |  |  |  |  |
| Should recommend data be collected as described Item 7 | 2.7.5 |  |  |  |  | 8 | (42) | 7 | (37) |  |  |  |  |
| Should recommend data be collected as described Item 8 | 2.7.5 |  |  |  |  | 10 | (53) | 5 | (26) |  |  |  |  |
| Moderate and Severe Myocarditis |  |  |  |  |  |  |  |  |  |  |  |  |  |
| Prefer moderate myocarditis Definition A vs. B | 1.4.1 | 12 | (55) | 9 | (41) |  |  |  |  |  |  |  |  |
| Prefer severe myocarditis Definition A vs. B | 1.4.3 | 13 | (59) | 7 | (32) |  |  |  |  |  |  |  |  |
| Should add “new onset” to arrhythmia in myocarditis definitions | 2.3.1 |  |  |  |  | 14 | (74) | 3 | (16) |  |  |  |  |
| Should recommend ECG for only those with clinical myocarditis | 2.3.2 |  |  |  |  | 11 | (58) | 6 | (32) |  |  |  |  |
| Should add “has adequate volume status” to severe Definition A | 2.4.1 |  |  |  |  | 13 | (68) | 3 | (16) |  |  |  |  |
| Prefer revised Definition A for severe myocarditis | 2.4.2 |  |  |  |  | 9 | (47) | 6 | (32) |  |  |  |  |
| Felt need for inotropes or ECHO evidence qualifies severe case | 3.4.2 |  |  |  |  |  |  |  |  | 13 | (72) | 3 | (17) |
| Prefer revised vs. newly revised Definition A for severe case | 3.4.1 |  |  |  |  |  |  |  |  | 7 | (39) | 7 | (39) |

* Question numbers consist of 3 integers separated by a period. The first integer refers to the round of inquiry; the second refers to a specific topic area, and the third is a subgroup of the second.

**Note: the total number of participants who agreed and disagreed to a specific question may not equal the column total of all active participants for that round because of non-responders, that is, participants were not obliged to respond to a specific question to proceed to
